# Nitrite impacts the survival of *Mycobacterium tuberculosis* in response to isoniazid and hydrogen peroxide

**DOI:** 10.1002/mbo3.126

**Published:** 2013-09-08

**Authors:** Amy Cunningham-Bussel, Franz C Bange, Carl F Nathan

**Affiliations:** 1 Department of Microbiology and Immunology, Weill Cornell Medical CollegeNew York, New York; 2 Graduate Program in Immunology and Microbial Pathogenesis, Weill Graduate School of Medical Sciences of Cornell UniversityNew York, New York; 3 Department of Medical Microbiology and Hospital Epidemiology, Medical School Hannover30625, Hanover, Germany

**Keywords:** Hydrogen peroxide, isoniazid, *Mycobacterium tuberculosis*, nitrate reductase, nitrite

## Abstract

When access to molecular oxygen is restricted, *Mycobacterium tuberculosis* (Mtb) can respire an alternative electron acceptor, nitrate. We found that Mtb within infected primary human macrophages in vitro at physiologic tissue oxygen tensions respired nitrate, generating copious nitrite. A strain of Mtb lacking a functioning nitrate reductase was more susceptible than wild-type Mtb to treatment with isoniazid during infection of macrophages. Likewise, nitrate reductase-deficient Mtb was more susceptible to isoniazid than wild-type Mtb in axenic culture, and more resistant to hydrogen peroxide. These phenotypes were reversed by the addition of exogenous nitrite. Further investigation suggested that nitrite might inhibit the bacterial catalase. To the extent that Mtb itself is the most relevant source of nitrite acting within Mtb, these findings suggest that inhibitors of Mtb's nitrate transporter or nitrate reductase could enhance the efficacy of isoniazid.

## Introduction

Nitrate (NO_3_^−^) and nitrite (NO_2_^−^) arise in the mammalian host from three major sources – dietary, bacterial, and host. Dietary nitrate is absorbed into the blood. Some is reduced by oral and gastrointestinal commensals, producing nitrite, which is also absorbed. Nitric oxide synthases (NOSs), including those that are constitutively expressed, produce nitric oxide, which autoxidizes to nitrate and nitrite. Even in healthy individuals, in whom relatively little of the high-output NOS isoform (iNOS) is expressed (Nicholson et al. 1996), nitrate is present in plasma at levels of 20–40 μmol/L (Lundberg and Weitzberg 2010). In patients with tuberculosis (TB), iNOS and other NOSs are expressed in granulomas (Choi et al. 2002; Lundberg et al. 2009).

At oxygen tensions of 1% or lower, Mtb itself can produce nitrite as it maintains adenosine-5'-triphosphate (ATP) synthesis and redox homeostasis by reducing an alternative electron acceptor, nitrate. Mtb constitutively expresses the *narGHJI* operon, which encodes Mtb's only functional nitrate reductase (Wayne and Sohaskey 2001; Sohaskey and Wayne 2003). However, transport of nitrate into the bacterium depends on transcription of a nitrate transporter encoded by *narK2*, a member of the dormancy survival (*DOS*) regulon (Sherman et al. 2001; Voskuil et al. 2003; Giffin et al. 2012). The *narK2* gene is expressed in axenically cultured Mtb only when oxygen levels fall below 1%. Induction of *narK2* markedly enhances mycobacterial nitrate respiration (Sohaskey and Wayne 2003). RNA transcripts from *narG,* which encodes a subunit of the nitrate reductase, and *narX,* an inactive fused nitrate reductase downstream of *narK2* in a shared operon, have been detected in lungs of patients with TB (Fenhalls et al. 2002; Sohaskey and Wayne 2003; Rachman et al. 2006), and RNA transcripts encoding the nitrate transporter, *narK2*, were elevated in the lungs of Mtb-infected mice (Shi et al. 2005). Thus, mycobacterial nitrate reduction likely occurs in the infected host.

Our laboratory recently converted our culture system for primary human macrophages from conventional gas phase conditions of 21% O_2_, 5% CO_2_ to 10% O_2_, 5% CO_2_ on the grounds that 10% O_2_ is physiologic for tissue macrophages (Vogt and Nathan 2011). To our surprise, infection of the macrophages in vitro with wild-type Mtb under 10% oxygen resulted in extensive accumulation of nitrite in the supernatant. Evidence will be presented elsewhere that macrophages were not the source of nitrite in this system. In contrast, as shown below, Mtb's NarG was the source, despite the nonhypoxic level of oxygen in the gas phase.

Wayne has proposed that nitrate respiration supports the establishment of a nonreplicating persistent state (NRP) in response to hypoxia (Wayne and Hayes 1998). We hypothesized that Mtb lacking the capacity for nitrate respiration might demonstrate an impaired entrance into NRP and therefore remain more susceptible to drugs that target replicating Mtb. The goal of the present study was to assess the contribution of nitrate respiration to Mtb's sensitivity to the drugs isoniazid (INH), ethambutol, streptomycin, and rifampicin. Compared to wild-type Mtb, *narG*-deficient Mtb, which is unable to produce nitrite, was selectively hypersusceptible to INH, as well as hyperresistant to hydrogen peroxide. INH requires oxidative activation by the mycobacterial catalase/peroxidase KatG in order to become cidal. The resultant isonicotinoyl radical adducts with cellular pyridine nucleotides and potently inhibits InhA, a member of type II fatty acid synthesis pathway involved in cell wall mycolic acid biosynthesis (Winder and Collins 1970; Rozwarski 1998). These findings imply that the action of INH on Mtb may be blunted by nitrite contributed by the host, the pathogen, or both.

## Methods

### Isolation and differentiation of primary human monocytes

Isolation of human monocytes and their differentiation into macrophages was as described (Vogt and Nathan 2011). In brief, heparinized peripheral blood was collected by venipuncture from healthy human donors who provided informed consent under an IRB approved protocol. Peripheral blood mononuclear cells were first isolated by centrifugation of whole blood over Ficoll-Paque (GE Healthcare, Uppsala, Sweden). The buffy coats were collected and monocytes were isolated by positive selection using magnetic beads conjugated to anti-CD14 antibodies (Miltenyi Biotec, Auburn, CA). Following isolation, the human monocytes were plated at a density of 500,000 cells/mL per well of a 96 well plate. The culture medium consisted of 60% Roswell Park Memorial Institute medium (RPMI) 1640, supplemented with 1% glutamax, 40% human plasma and granulocyte-macrophage colony-stimulating factor (GM-CSF) and tumor necrosis factor alpha (TNFα) (0.5 ng/mL each). Thirty percent of the total culture volume was replaced with fresh medium and cytokines every 3–4 days. Replacing fresh medium was conducted as rapidly as possible in room air before the cells were placed back in the low oxygen incubator. Differentiation of the monocytes was conducted over 2 weeks in our standard medium at 10% O_2_ and 5% CO_2_ at 37°C in a humidified atmosphere in a chamber flushed with N_2_ under the control of a PRoOX sensor and ProCO_2_ regulator (BioSpherix, Lacona, NY). They were then activated with interferon gamma (IFNγ) (5 ng/mL) before infection with *Mycobacterium tuberculosis* (Mtb) the following day at the desired multiplicity of infection (MOI).

### Preparation of Mtb and infection of human macrophages with Mtb

*Mycobacterium tuberculosis* H37Rv was grown in Middlebrook 7H9 broth supplemented with 0.2% glycerol, 0.5% bovine serum albumin (BSA), 0.2% dextrose, and 0.085% NaCl with 0.05% Tween 80. The *narG*-deficient and complemented strains were generated as described (Stermann et al. 2003). Cultures were started with 1 mL stock originally frozen at −80°C in log phase and then grown over 4–5 days to optical densities (OD) of 0.5–1.25 before the start of an experiment. A single-cell suspension was generated by centrifugation at 120 g for 10 min. For macrophages infection, roughly 200 × 10^6^ bacteria were centrifuged, in order to be able to observe a pellet. The 7H9 was completely removed and the cells were washed with phosphate buffered saline (PBS). The bacteria were then resuspended in culture medium and the desired number of Mtb was added to the macrophage culture.

Certain experiments required that we remove the nitrate-containing medium from the cell culture. In this case, 1 day prior to infection the cells were washed with room temperature PBS three times and a low-nitrate formulation of Dulbecco's Modified Eagle Medium (DMEM) with 10% human plasma was added along with 0.5 ng/mL GMCSF and TNFα and 5 ng/mL IFNγ.

### Measurement of nitrite

Nitrite levels produced in the coculture supernatants of infected macrophages were measured by the Griess assay. Briefly, 100 μL of supernatant was removed and to this were added 50 μL of 2% sulfanilamide with 5% phosphoric acid and 50 μL of 0.2% N-1-napthylethylenediamine dihydrochloride. Nitrite standards were prepared in the same medium used to culture the macrophages. Absorbance was measured at 550 nm immediately after addition of the reagents to the supernatant samples.

### Treatment of infected macrophages

Macrophages were incubated with Mtb at the indicated MOI for 4–5 h under 10% oxygen. The supernatant was removed and the macrophages were washed three times with room temperature PBS to remove extracellular bacteria. Fresh medium was added to the cells, followed by the addition of nitrite, nitrate, and/or an antimycobacterial drug such as INH. The cultures were then returned to the low oxygen incubator and incubated for a subsequent 3 days with the desired compound. Drugs were freshly dissolved before each experiment in dimethyl sulfoxide (DMSO), except for streptomycin, which was dissolved in water.

### Colony forming units determination from infected macrophages

Following incubation with Mtb for 3 days, the macrophages were inspected by microscopy to ensure continued confluence and adherence to the well. The supernatant was collected and the cells were gently washed three times with room temperature PBS to wash away remaining compounds. Following the wash steps, the cells were again visualized to confirm that the monolayer was intact and then lysed by incubation in 0.5% Triton X-100 (Sigma-Aldrich, St. Louis, MO) for 10 min at 37°C. The lysate was serially diluted in 0.1% Triton X-100 and plated on Middlebrook 7H11 agar with 10% oleic acid–albumin–dextrose–catalase (OADC) enrichment (Becton Dickinson, Franklin Lakes, NJ) supplemented with 0.5% glycerol. Colonies were enumerated following 3 weeks of culture at 37°C, in 21% O_2_, 5% CO_2_.

### Treatment of Mtb in axenic culture

*Mycobacterium tuberculosis* was added at the indicated OD to 96 well plates in 7H9 broth with or without 5 mmol/L nitrate or the indicated concentration of nitrite, supplemented with 10 ADNaCl (0.2% glycerol, 0.5% BSA, 0.2% dextrose, 0.085% NaCl) and 0.05% Tween 80. The indicated antibiotic was added immediately and the plate was incubated in 1% oxygen for 3 days or at 21% oxygen for 5 days, as indicated. The Mtb was resuspended, serially diluted in 0.1% Triton-X 100 (Sigma-Aldrich), and plated for colony forming units (CFU) as above. Alternatively, for OD measurements, the Mtb was resuspended in the culture medium and the absorbance was read at 580 nm on the fifth day of incubation with the indicated compound. In the case of exposure to hydrogen peroxide, the plate was incubated in 1% oxygen for 8 h with or without 1 mmol/L nitrate or the indicated concentration of nitrite. At this point, the indicated concentration of hydrogen peroxide was added and the cells were incubated overnight at 1% oxygen before plating for CFU.

### KatG western blot and measurement of KatG dependent oxidation of INH

A minimum of 4 × 10^9^ bacteria per condition were suspended in 7H9, 10% ADNaCl at an OD of 0.2 in the presence or absence of 2.5 mmol/L nitrite. Cultures were incubated for 1 day in 1% oxygen. The cultures were collected by centrifugation and resuspended in 200 μL of lysis buffer containing 50 mmol/L NaPO_4_, 1 mM phenylmethylsulfonyl fluoride (PMSF), and 4× complete protease inhibitor cocktail (Roche, Basel, Switzerland). The cells were lysed in a bead-beating homogenizer with silica beads three times with intermittent incubation on ice. The lysate was collected and filter sterilized and concentrated at 4°C to ∼100 μL using Amicon Ultra-0.5 mL centrifugal filters from Millipore (Billerica, MA) with a molecular weight cutoff of 3 kDa. Protein concentration was measured using the Bio-Rad (Hercules, CA) assay and equal amounts of protein from each sample were loaded into 7.5% reducing gels. After electrophoresis, proteins were transferred onto a polyvinylidene difluoride membrane, blocked with Odyssey blocking buffer from LI-COR Biosciences (Lincoln, NE) and stained with anti-KatG antibody (obtained through BEI Resources, NIAID, NIH: Monoclonal Anti-*Mycobacterium tuberculosis* KatG, gene Rv1908c, clone IT-57 [CDA4] [culture supernatant], NR-13793) and anti-DlaT antibody (1:1000) and then visualized with fluorophore-coupled secondary antibodies (Venugopal et al. 2011). To test the degree of KatG-dependent oxidation of INH, 50 μg of total cell lysate was loaded into 7.5% nonreducing gels. After electrophoresis, the gel was incubated at room temperature for 15–30 min in 50 mL of NaPO_4_ buffer containing 62 mg INH, and 2 mmol/L nitroblue tetrazolium (NBT) at pH 7 to which hydrogen peroxide was added to a final concentration of 800 μmol/L (Saint-Joanis et al. 1999).

### Quantitative polymerase chain reaction of RNA from Mtb growing in macrophages or broth culture

Five million human macrophages were cultured per T25 flask in 10% oxygen and infected with wild-type, *narG-*deficient, or the complemented strains of Mtb at an MOI of 40 for 10 h. Supernatant was removed and the monolayer was washed two times with room temperature PBS. Trizol (2 mL) was added to each flask and the cells were detached using a rubber policeman. For broth cultures, at least 2.5 × 10^9^ bacteria per condition were incubated in 1% oxygen for 3 days with or without 2.5 mmol/L nitrite and 0.1 μg/mL INH. Then an equal volume of 5 mol/L GTC buffer containing 5M guanidine thiocyanate, 25 mmol/L sodium citrate, 20 mmol/L N-lauryl-sarcosine, 0.7% v/v β- mercaptoethanol was added. The bacteria were pelleted by centrifugation and 1 mL of Trizol was added per sample. The suspension was beaten with silica beads, three times. RNA was extracted using an RNeasy kit (Qiagen, Venlo, Limburg, Netherlands) in accordance with the manufacturer's instructions with the exception that off column DNAse digestion was performed for 2 h at 37°C. To generate cDNA, total RNA (900 ng) was reverse transcribed using the GeneAmp RNA polymerase chain reaction (PCR) Kit (Life Technologies, Carlsbad, CA). Quantitative RT-PCR was performed using gene-specific primers (Life Technologies) and the SuperScript III Platinum Two Step real-time quantitative PCR (qRT-PCR) Kit (Life Technologies) with a 7900HT Fast Real Time PCR System (Life Technologies). Each experiment contained experimental triplicates. All reported values were within the linear range of the primers and the experimental results were normalized to 16S rRNA values.

### Statistical analysis

Statistical analysis was performed as indicated in the figure legends using the standard statistical software Prism version 5.0f for Macintosh, GraphPad Software, San Diego, CA.

## Results

### Nitrate respiration enhanced mycobacterial resistance to isoniazid within primary human macrophages

**Figure 1:**
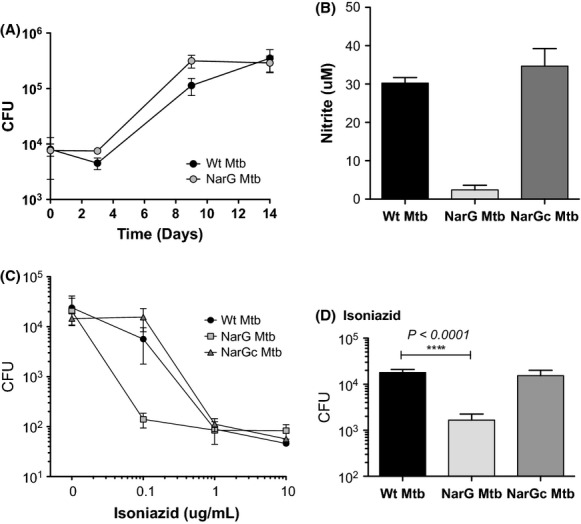
Effect of nitrate respiration on mycobacterial resistance to isoniazid within primary human macrophages. (A) Survival of wild-type and *narG-*deficient (NarG) Mtb within macrophages (MOI: 0.1, corresponding to 10,000 bacteria). (B) Nitrite levels arising in the supernatant of macrophages 3 days after infection with wild-type Mtb (Wt Mtb), NarG Mtb, and the complemented strain (NarGc Mtb) (MOI: 5). (C) Susceptibility of Mtb to INH within infected macrophages (MOI: 0.1) over 3 days. Means ± SEM from one experiment representative of three independent experiments using cells from different donors. (D) As in (C), pooling results from the three donors (means ± SEM) at a single INH concentration administered to the culture for 3 days (0.1 μg/mL). The p value was determined by an unpaired *t-*test.

We observed no difference in survival of wild-type (Wt Mtb) and *narG*-deficient Mtb (NarG Mtb) during infection of primary human macrophages under 10% O_2_ and 5% CO_2_ (Fig. 1A). During the course of infection, large quantities of nitrite arose in the supernatant of macrophages infected with wild-type Mtb, which was not observed in the supernatants of macrophages infected with *narG*-deficient Mtb (Fig. 1B). To test the role of nitrate reduction on the susceptibility of Mtb to antibiotics, we infected macrophages with wild-type, *narG*-deficient, and complemented strains (NarGc Mtb) and treated the cultures with drugs. Mtb deficient in *narG* was hypersusceptible to INH, with a 10-fold decrease in the MBC99 compared to wild type (Fig. 1C–D). In contrast, *narG* made no observable difference to the antibacterial actions of streptomycin, rifampicin, or ethambutol on Mtb within macrophages (Fig. S1A–F).

### Addition of nitrite reversed the hypersusceptibility of *narG*-deficient Mtb to isoniazid

**Figure 2:**
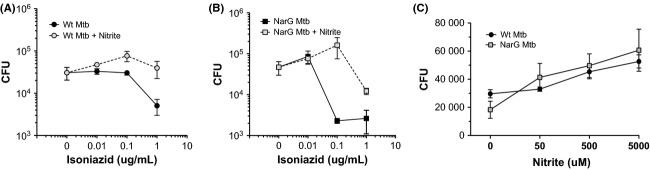
Impact of exogenous nitrite on the hypersusceptibility of narG-deficient Mtb to isoniazid. (A, B) The effect of nitrite on the survival of (A) wild-type or (B) *narG*-deficient (NarG) Mtb within INH-treated infected macrophages (MOI: 0.1). The standard culture medium was replaced with low-nitrate medium. Where indicated by the dashed lines, exogenous sodium nitrite (final concentration 1 mmol/L) was added to the culture medium along with the indicated concentrations of INH. CFU were harvested 3 days later. (C) Survival of wild-type and *narG*-deficient (NarG) Mtb within macrophages treated with increasing concentrations of nitrite in the presence of a single concentration of INH (0.1 μg/mL). The standard culture medium was replaced with low-nitrate medium and exogenous sodium nitrite was added at the indicated final concentrations. Each result is representative of at least two independent experiments (means ± SEM).

The hypersusceptibility of *narG-*deficient Mtb to INH may have resulted from loss of the metabolic energy generated by nitrate reduction or from failure to produce nitrite. To distinguish between these possibilities, we added exogenous nitrite to the medium of infected macrophage cultures treated with INH. Because the standard culture medium consisted of 40% human plasma and 60% RPMI 1640, it contained high concentrations of nitrate. Therefore, to limit the accumulation of nitrite produced by wild-type Mtb, we replaced the medium immediately prior to infection with a low-nitrate medium (to minimize perturbation we refrained from washing the cells thoroughly; thus, medium exchange was not complete). Addition of exogenous nitrite enhanced bacterial survival in the presence of INH in a dose-dependent manner (Fig. 2A–C). That this effect was less striking in the case of wild-type Mtb was likely due to residual nitrate in the medium, allowing for low-level nitrite production (Fig. 2A).

### Mtb deficient in *narG* Mtb was hypersusceptible to isoniazid in axenic culture

**Figure 3:**
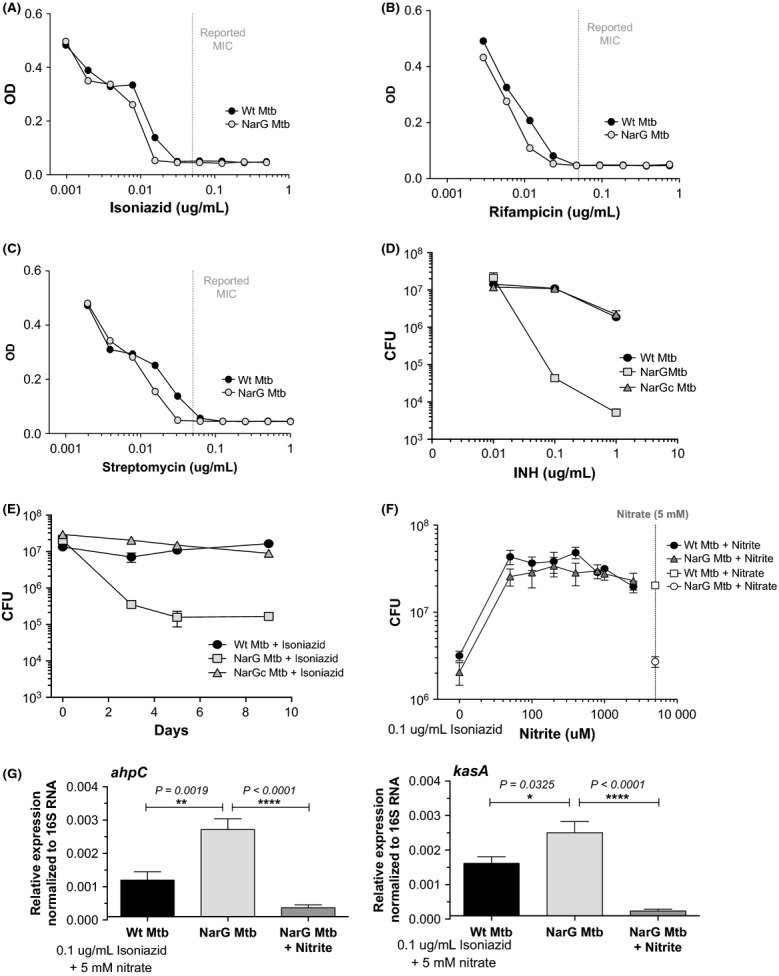
Impact of *narG* deficiency on susceptibility of Mtb to isoniazid in axenic culture. (A–C) Survival of wild-type and *narG-*deficient Mtb in the presence of the indicated concentrations of (A) isoniazid, (B) rifampicin, or (C) streptomycin in 21% oxygen over 5 days, initial (OD 0.1). Published MICs are provided for reference (Wanger and Mills 1996). (D, E) Survival of wild-type, *narG-*deficient (NarG), and complemented (NarGc) strains cultured in 1% oxygen (D) for 3 days with the indicated concentrations of INH or (E) as a function of time over 9 days with 0.1 μg/mL INH. (F) Survival of wild-type and *narG*-deficient Mtb incubated with increasing concentrations of exogenous nitrite or one fixed dose of nitrate, as indicated, over 3 days in 1% oxygen with 0.1 μg/mL INH. Results for (A–F) are means ± SEM representative of two independent experiments. (G) Expression of transcripts of *ahpC* and *kasA* by wild-type and *narG-*deficient Mtb treated with 0.1 μg/mL INH and 5 mmol/L nitrate for 16 h at 1% oxygen relative to 16S RNA. Where indicated, 2.5 mmol/L nitrite was also added. Bars indicate means ± SEM from three independent experiments analyzed by unpaired *t-*tests.

*Mycobacterium tuberculosis* respires nitrate when infecting human macrophages under 10% oxygen, but in axenic culture, as noted, nitrate respiration is barely detectable until oxygen is lowered to 1% or less. This implied that within macrophage phagosomes, the functional level of oxygen perceived by Mtb was far lower than the level present in the gas phase. The nitrate reductase assay is routinely used in clinical laboratories to identify drug resistant Mtb (Angeby et al. 2002). Although the assay is carried out under room air (21% oxygen), nitrate reductase activity is measured at the end of a prolonged incubation period, which may indicate that the medium in which Mtb is incubated becomes hypoxic. Alternatively, while aerobic cultures of Mtb do not express the nitrate transporter, *narK2*, nitrate may gradually diffuse across the plasma membrane and be reduced to nitrite. In order to test the role of nitrate respiration in Mtb's susceptibility to INH in the absence of macrophages, we cultured wild-type and *narG*-deficient Mtb in 21% oxygen or 1% oxygen. No measurable survival differences were observed for the two strains in room air in response to isoniazid, streptomycin, or rifampicin (Fig. 3A–C). Under 1% oxygen, however, the survival of *narG-*deficient INH-treated Mtb dropped by 2–3 log_10_ as compared to wild-type and complemented strains (Fig. 3D), which was due to the induction of the nitrate reductase system. Next, we examined survival as a function of time rather than concentration. A single application of INH led to a drop of 2 log_10_ in CFU of the *narG*-deficient strain over 9 days, while the CFU of the wild-type and complemented strains remained constant (Fig. 3E). Exogenous nitrite enhanced the survival of the *narG*-deficient and wild-type strains cultured in the absence of nitrate and treated with INH (Fig. 3F).

Following INH treatment and its subsequent activation by KatG, Mtb induces the expression of *kasA* and *ahpC* (Wilson et al. 1999). The *narG-*deficient strain demonstrated greater induction of *kasA* and *ahpC* RNA transcripts than the wild-type strain in response to 0.1 μg/mL INH, and induction was attenuated following treatment with nitrite (Fig. 3G). INH requires oxidative activation by the mycobacterial catalase/peroxidase KatG in order to become bioactive. These results suggest that when presented with the same concentration of INH under conditions that could sustain nitrate respiration by wild-type Mtb, *narG*-deficient Mtb converted a larger proportion of the INH prodrug to its active form than wild-type Mtb. This implied that the production of nitrite during respiration of nitrate by wild-type Mtb might partially inhibit KatG.

### *narG*-deficient Mtb was not hypersusceptible to treatment with ethionamide

**Figure 4:**
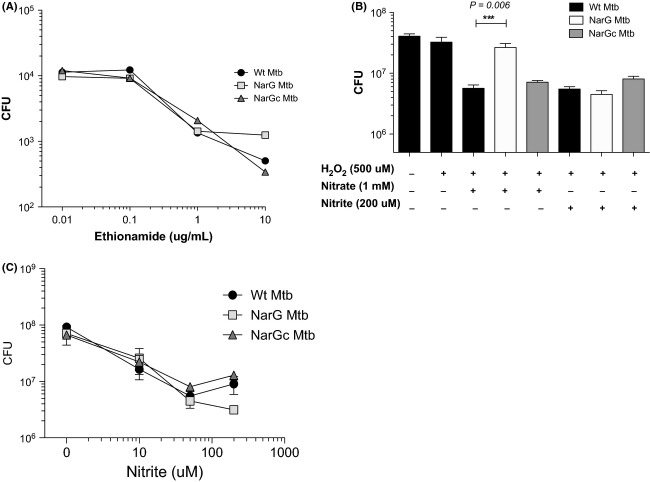
Impact of *narG* deficiency on susceptibility of Mtb to ethionamide and hydrogen peroxide. (A) Survival of wild-type and *narG*-deficient Mtb within infected primary human macrophages (MOI: 0.1) treated with the indicated concentrations of ethionamide for 3 days at 10% oxygen. (B) Survival of Mtb in axenic culture treated with nitrate or nitrite for 8 h in 1% oxygen, to allow for nitrate respiration to occur, and then treated with hydrogen peroxide (500 μmol/L) and incubated in 1% oxygen overnight (initial OD580: 0.1). These are pooled data from two independent experiments and were analyzed by an unpaired *t-*test. (C) Survival of wild-type, *narG*-deficient (NarG Mtb), and complemented strains (NarGc Mtb) (initial OD580: 0.1) following treatment with both hydrogen peroxide (500 μmol/L) and increasing concentrations of nitrite as indicated. Individual experiments representative of at least two independent experiments. Bars indicate means ± SEM.

To further investigate the role of KatG in mediating the hypersusceptibility of *narG*-deficient Mtb to INH, we infected human macrophages with Mtb and treated the cultures with ethionamide (ETH). ETH and INH are close chemical analogs. Both are prodrugs that when activated inhibit the same target, InhA. However, ETH is activated by the monoxygenase EtaA rather than by KatG (Baulard et al. 2000; DeBarber et al. 2000). Survival of wild-type and *narG*-deficient Mtb treated with ETH within infected human macrophages did not differ (Fig. 4a). Therefore, the INH susceptibility of *narG-*deficient Mtb is unlikely to be due to differential dependence on InhA and may instead be due to differences in the activation of INH by KatG.

### Nitrate reduction enhanced the lethality of hydrogen peroxide

Levels of KatG mRNA determined by qRT-PCR and KatG protein detected by western blot did not differ among wild-type, *narG*-deficient, and complemented strains of Mtb (Fig. S2B–C). Moreover, we were unable to measure differences in the extent of KatG-dependent oxidation of INH via NBT staining of polyacrylamide gel electrophoregrams of lysates obtained from the three strains (data not shown). Nonetheless, to help assess whether the catalytic activity of KatG might differ between wild-type and *narG-*deficient Mtb, the strains were incubated for several hours with nitrite or nitrate at 1% oxygen to allow for nitrate respiration and then treated with hydrogen peroxide. We reasoned that if the activity of KatG were attenuated by nitrite, the wild-type and complemented strains might be more susceptible to hydrogen peroxide than *narG-*deficient Mtb. In accord with this hypothesis, *narG-*deficient Mtb was more resistant to hydrogen peroxide than the other two strains (Fig. 4B). The enhanced mycobactericidal effect of hydrogen peroxide on the wild type and complemented strains was also observed following the addition of increasing concentrations of exogenous nitrite in the absence of nitrate (Fig. 4C and S2B).

## Discussion

Herein, we report that deficiency of *narG* increases the susceptibility of Mtb to INH and decreases its susceptibility to hydrogen peroxide. Prior to this work, the knockout of only one gene, *cydC*, had been reported to increase the susceptibility of Mtb to INH (Dhar and McKinney 2010). The mechanism in the present study most likely involves nitrite-mediated impairment of the activity of KatG that takes place when wild-type Mtb reduces nitrate to nitrite, which is absent in *narG*-deficient Mtb. Although nitrate is usually omitted from mycobacterial culture media, it is a physiologic constituent of human body fluids. Although Mtb does not significantly reduce nitrate to nitrite in axenic culture unless the culture is hypoxic, we found that Mtb does reduce nitrate to nitrite when cultured within macrophages at physiologic oxygen tensions. Thus, phenotypic INH resistance arising from Mtb's nitrate respiration is likely to occur in the Mtb-infected host.

Interactions of catalase and other peroxidases with nitrite are complex (Klebanoff 1993; van der Vliet et al. 1997; Eiserich et al. 1998; Battistuzzi et al. 2010). When we incubated Mtb with nitrite, lysed the cells, separated the proteins by nonreducing polyacrylamide gel electrophoresis and incubated the gel with INH and a tetrazolium dye as described (Saint-Joanis et al. 1999), we did not see impairment in the activity of catalase compared to lysates from Mtb not exposed to nitrite (not shown). However, the biochemical environment within intact mycobacteria differs markedly from that of gels in room air, and tetrazolium is not a physiologic oxidant for INH. Moreover, *katG* mutations that cause INH resistance do not necessarily impact the rate of INH-NAD adduct formation by KatG relative to wild-type Mtb, pointing to the potential for dissociation between the functional effects of KatG mutations and the results of biochemical assays for KatG activity (Musser et al. 1996; Wengenack et al. 2004; Ghiladi et al. 2005; Cade et al. 2010).

The following evidence supports the hypothesis that nitrite interfered with the INH-activating function of catalase in the intact mycobacterium: Mtb deficient in *narG* was both more susceptible to INH and more resistant to hydrogen peroxide than wild-type Mtb. Second, *narG-*deficient Mtb was not hypersusceptible to treatment with ETH. Third, compared to wild-type and complemented strains, *narG-*deficient Mtb demonstrated higher expression of *ahpC* and *kasA*, two genes induced by treatment with INH, and their expression was reduced by the addition of nitrite to the cell culture. Taken together, these results suggest that Mtb lacking *narG* achieves greater KatG-mediated activation of INH than wild-type Mtb. Lastly, in agreement with this hypothesis, *narG*-deficient Mtb better resisted treatment with hydrogen peroxide. The mutation of genes other than *katG* can also cause INH resistance. For example, mutations that impair the catalytic activities of Ndh, a type II NADH dehydrogenase, and MshA and MshC*,* which contribute to mycothiol biosynthesis, can cause INH resistance. However, inactivating mutations in any one of these genes also cause ETH resistance (Vilchèze et al. 2005; Hazbon et al. 2006; Vilcheze et al. 2011a), which was not observed in this study.

Nitric oxide or nitrite at low pH, which generates nitric oxide (Lundberg et al. 2008), can potentiate the bactericidal activity of hydrogen peroxide toward *Escherichia coli* (Klebanoff 1993; Pacelli et al. 1995; Woodmansee and Imlay 2003; Lundberg and Weitzberg 2010). However, to our knowledge, this is the first report that nitrite at neutral pH can synergistically enhance the bactericidal action of hydrogen peroxide. Production of hydrogen peroxide by phagocytic cells is an important component of host antibacterial defense (Nathan and Shiloh 2000). Mycobacterial nitrate respiration may enhance the lethality of host reactive oxygen species in vivo. This may result from inhibition of KatG by nitrite or from inhibition of a variety of antioxidant defense proteins, including KatG, by products more reactive than nitrite to which nitrite can give rise. In *E. coli*, nitrate reductase is an important source of species reactive enough to nitrosylate proteins, as evidenced by an 80% reduction in protein S-nitrosylation in *narG-*deficient as compared to wild-type bacteria (Ralt et al. 1988; Corker 2003; Seth et al. 2012). Rhee et al. (2005) identified 29 mycobacterial proteins that were S-nitrosylated when Mtb was treated with nitrite at low pH, including KatG. In addition, heme peroxidases such as KatG oxidize nitrite to more reactive species, such as nitrogen dioxide (^•^NO_2_) and nitryl chloride (NO_2_Cl) (Klebanoff 1993; van der Vliet et al. 1997; Eiserich et al. 1998; Battistuzzi et al. 2010).

INH is widely reported to lose mycobactericidal activity when Mtb is cultured in conditions that prevent it from replicating. However, when the oxygen in an axenic, nitrate-containing culture of Mtb was reduced below 1%, *narG*-deficient Mtb, which was not replicating, became highly INH sensitive. We confirmed that effect and demonstrated its reversal with exogenous nitrite. To the extent that a relevant source of nitrite for Mtb residing in a macrophage in the human host may be Mtb itself, the question arises whether the cidality of INH for nonreplicating *narG*-deficient Mtb could be phenocopied in nonreplicating wild-type Mtb by an inhibitor of Mtb's nitrate transporter or nitrate reductase. If so, such agents might significantly reduce the time required to cure latent TB with INH or active TB with INH-containing regimens, reducing the incidence of emergent drug resistance associated with incomplete treatment (van den Boogaard et al. 2009; Zumla et al. 2013). InhA is essential to aerobically and perhaps also hypoxically cultured mycobacteria as two compounds that targeted both InhA as well as fatty acid synthase type 1 were cidal to nonreplicating Mtb (Vilcheze et al. 2011a,b). Nevertheless, it remains unclear whether the reduced survival of *narG-*deficient Mtb treated with INH resulted only from the inactivation of InhA or by inhibition of additional mycobacterial target(s). A mechanistic understanding of the hypersusceptibility of *narG-*deficient Mtb to INH may reveal new INH target(s) amenable to inhibition by other compounds.

## Supporting Information

**Figure S1.** (A–C) Survival of wild-type, *narG-*deficient (NarG), and complemented strains (NarGc) within macrophages (MOI: 0.1, corresponding to 10,000 bacteria) treated with the indicated concentrations of (A) rifampicin (B) streptomycin, or (C) ethambutol over 3 days in 10% oxygen. The results shown in A–C are single experiments that are representative of at least two independent experiments conducted using cells isolated from at least two distinct human donors. (D–F) contain the pooled CFU data from at least two independent experiments conducted with cells from at least two individual human donors shown for one drug concentration as follows: (D) rifampicin (0.2 μg/mL), (E) streptomycin (0.6 μg/mL), or (F) ethambutol (0.1 μg/mL). Bars indicate means ± SEM.

**Figure S2.** (A) The survival of axenic cultures of Mtb (OD 0.1) following treatment with the indicated concentration of hydrogen peroxide at 1% oxygen incubated overnight with 1 mmol/L nitrite. This is representative of two independent experiments. (B) The mRNA expression of *katG* by wild-type, *narG* -deficient (NarG), and the complemented (NarGc) strains within infected human macrophages following incubation for 3 days at 10% oxygen. The given experiment is the pooled result of two independent experiments. (C) Axenic cultures of wild-type, *narG*-deficient, and the complemented strains of Mtb were incubated over 1 day at 1% oxygen and lysed to collect total cell protein. Individual proteins from the total cell lysate were separated by native gel electrophoresis and KatG and the housekeeping gene DLAT were visualized by fluorophore-coupled antibody staining. This is representative of three independent experiments. Bars indicate means ± SEM.
