# Supplementary material for: Nitrite impacts the survival of Mycobacterium tuberculosis in response to isoniazid and hydrogen peroxide
Source: Microbiologyopen. 2013 Sep 8;2(6):901–11. doi: 10.1002/mbo3.126 (PMC3892337; doi:10.1002/mbo3.126)

Figure 1

INFECTED HUMAN MACROPHAGES

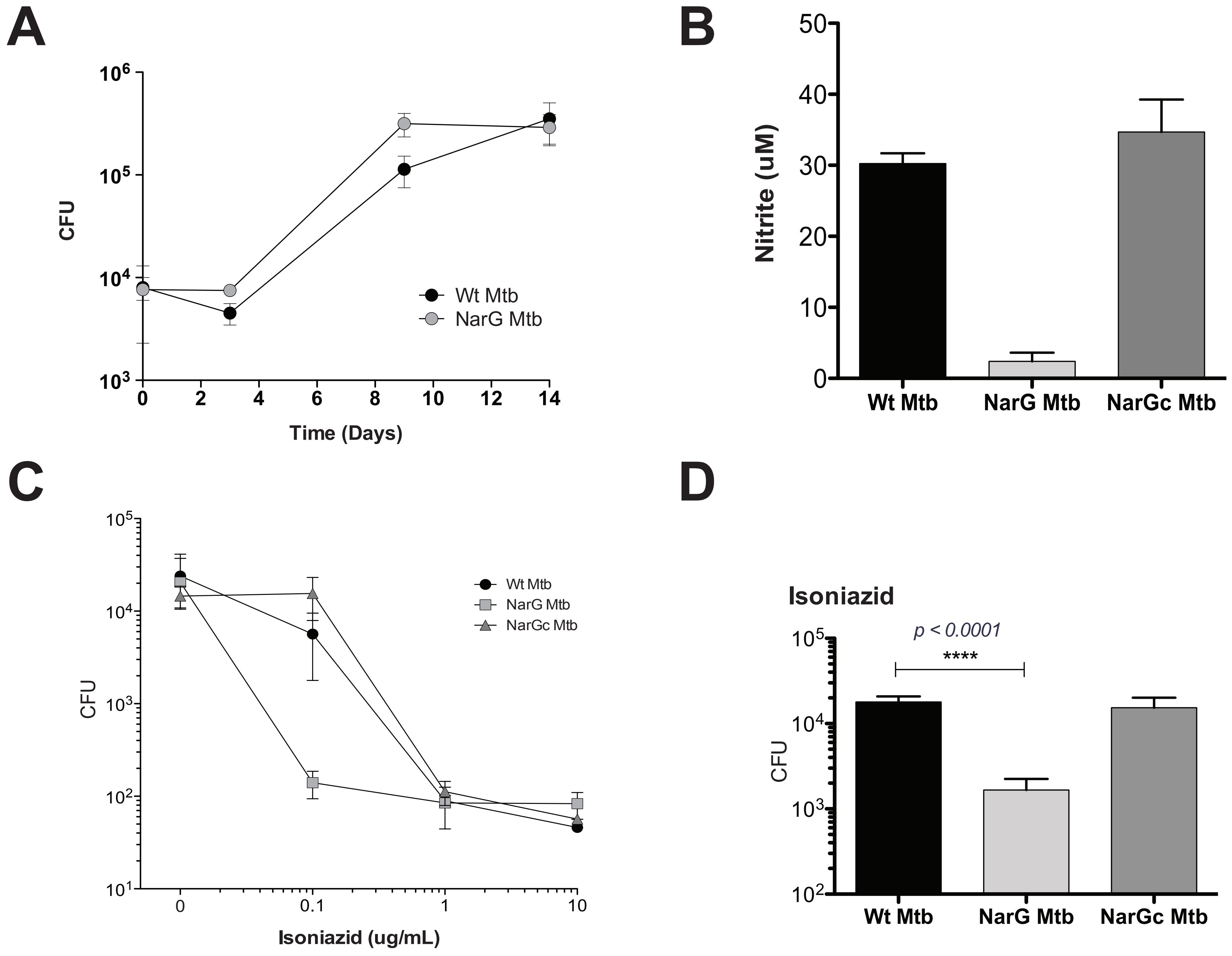

Figure 2

Infected human macrophages

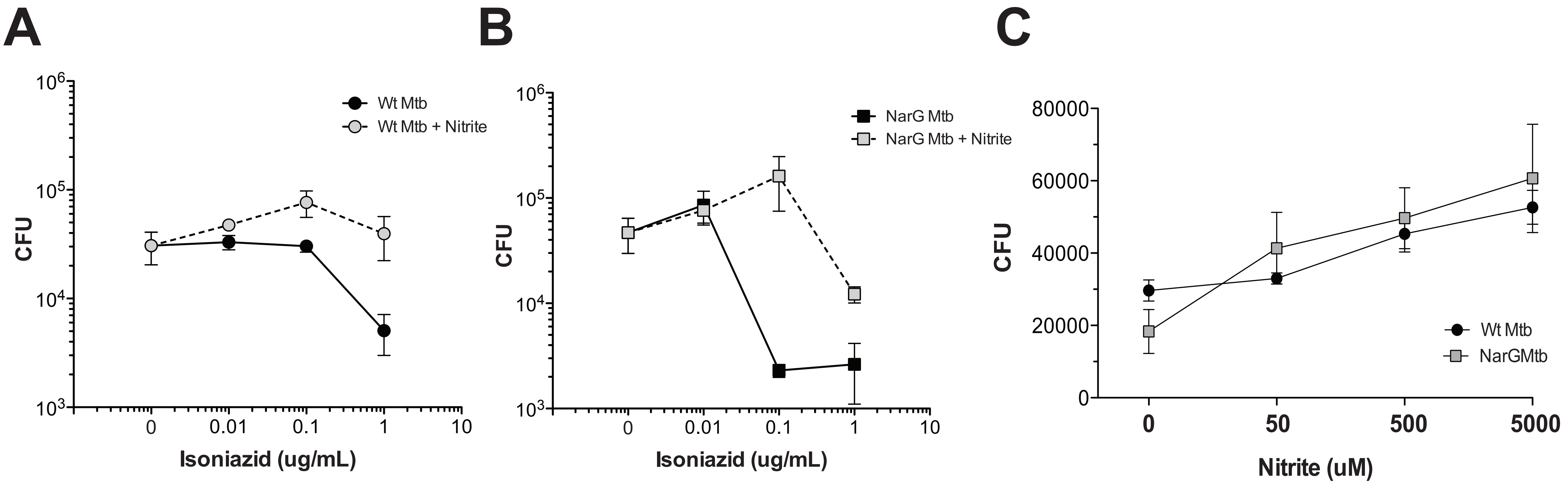

Figure 3

Broth culture of Mtb in the absence of human macrophages

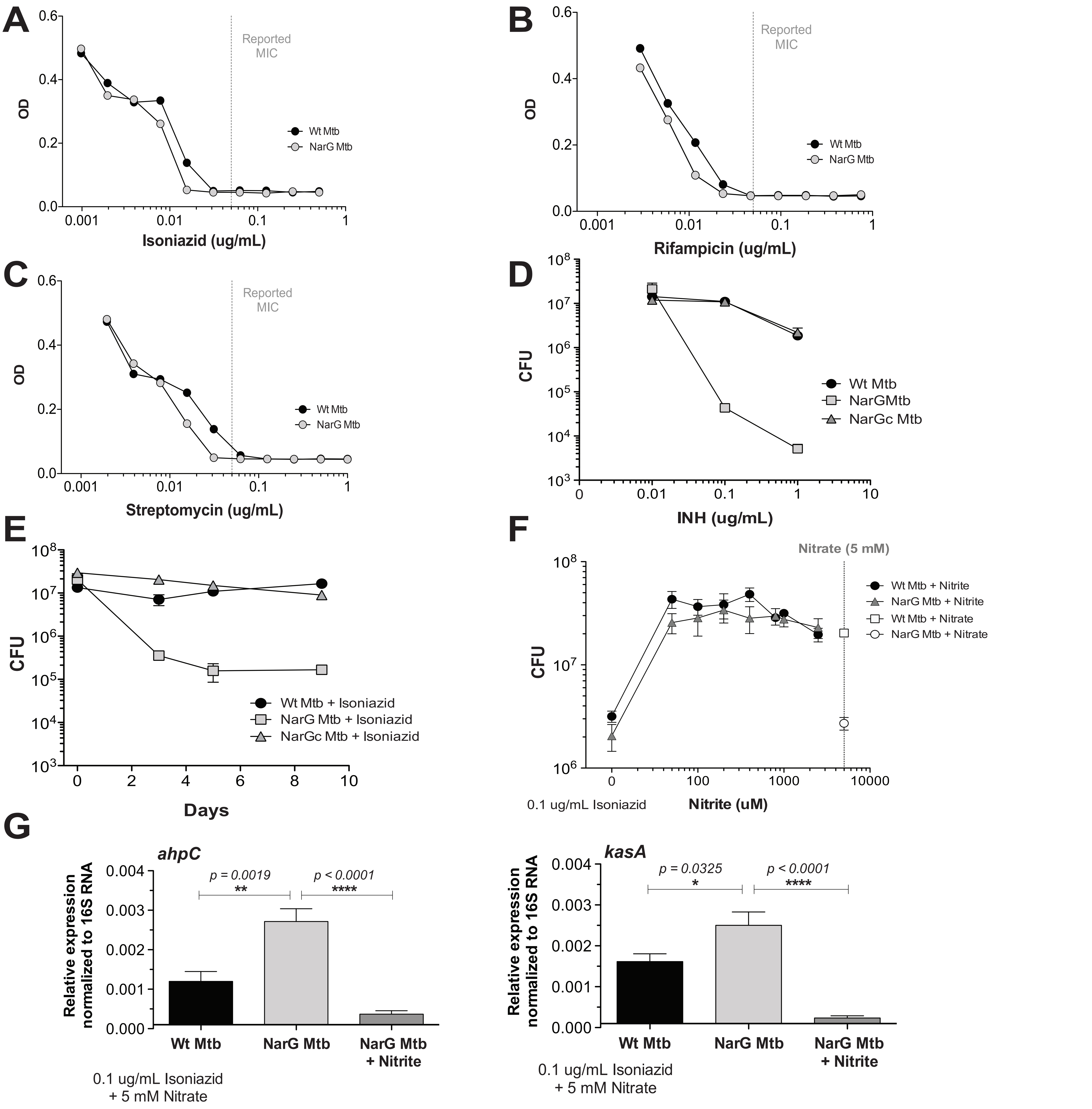

Figure 4

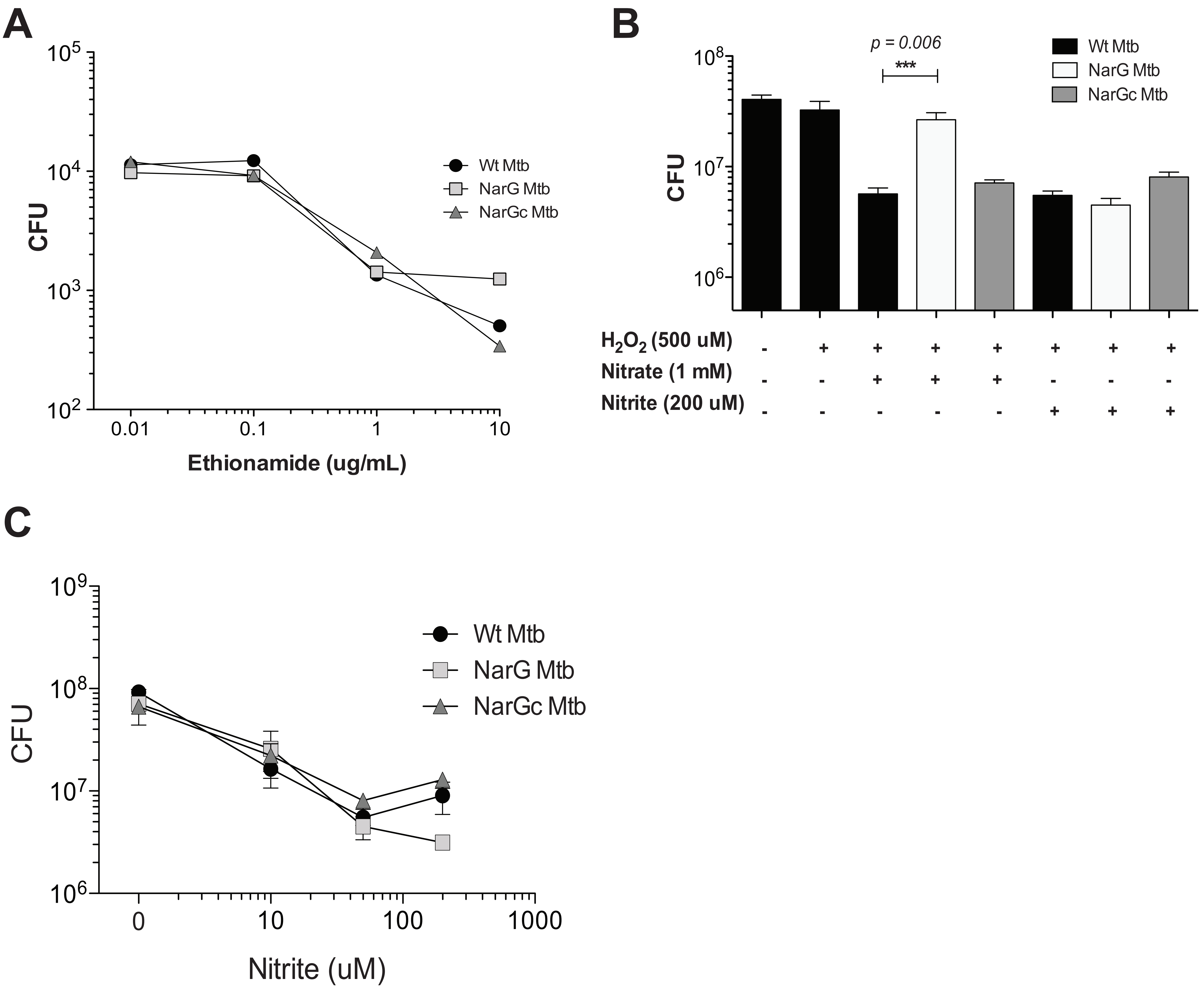

A
CFU
10^5
10^4
10^3
10^2
0.01
0.1
1
10
Ethionamide (ug/mL)
Wt Mtb
NarG Mtb
NarGc Mtb
B
p = 0.006
***
CFU
10^8
10^7
10^6
Wt Mtb
NarG Mtb
NarGc Mtb
H2O2 (500 uM) - + + + + + + +
Nitrate (1 mM) - - + + + - - -
Nitrite (200 uM) - - - - - + + +
C
CFU
10^9
10^8
10^7
10^6
0
10
100
1000
Nitrite (uM)
Wt Mtb
NarG Mtb
NarGc Mtb

Supplementary Figure 1

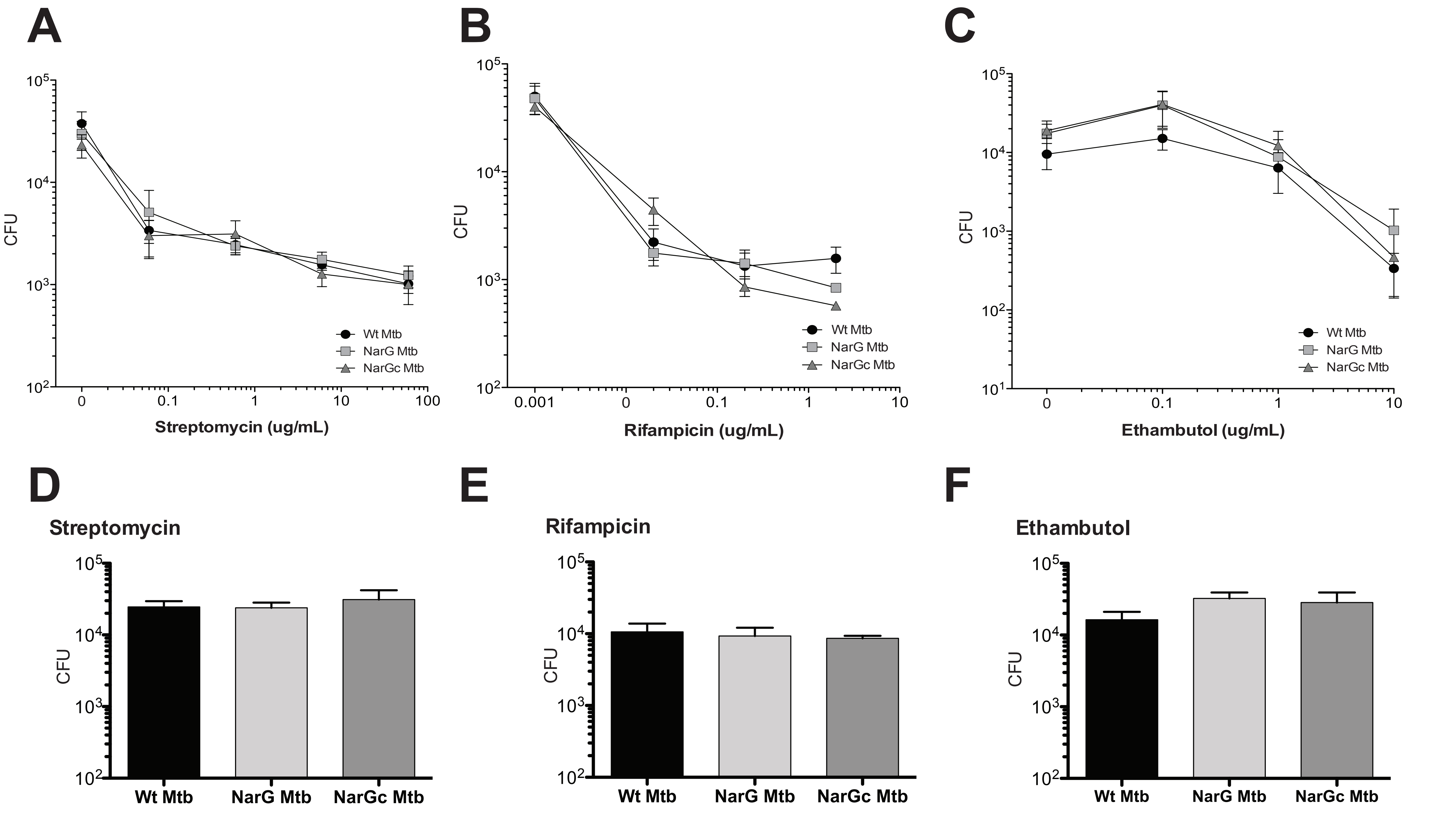

Supplementary Figure 2

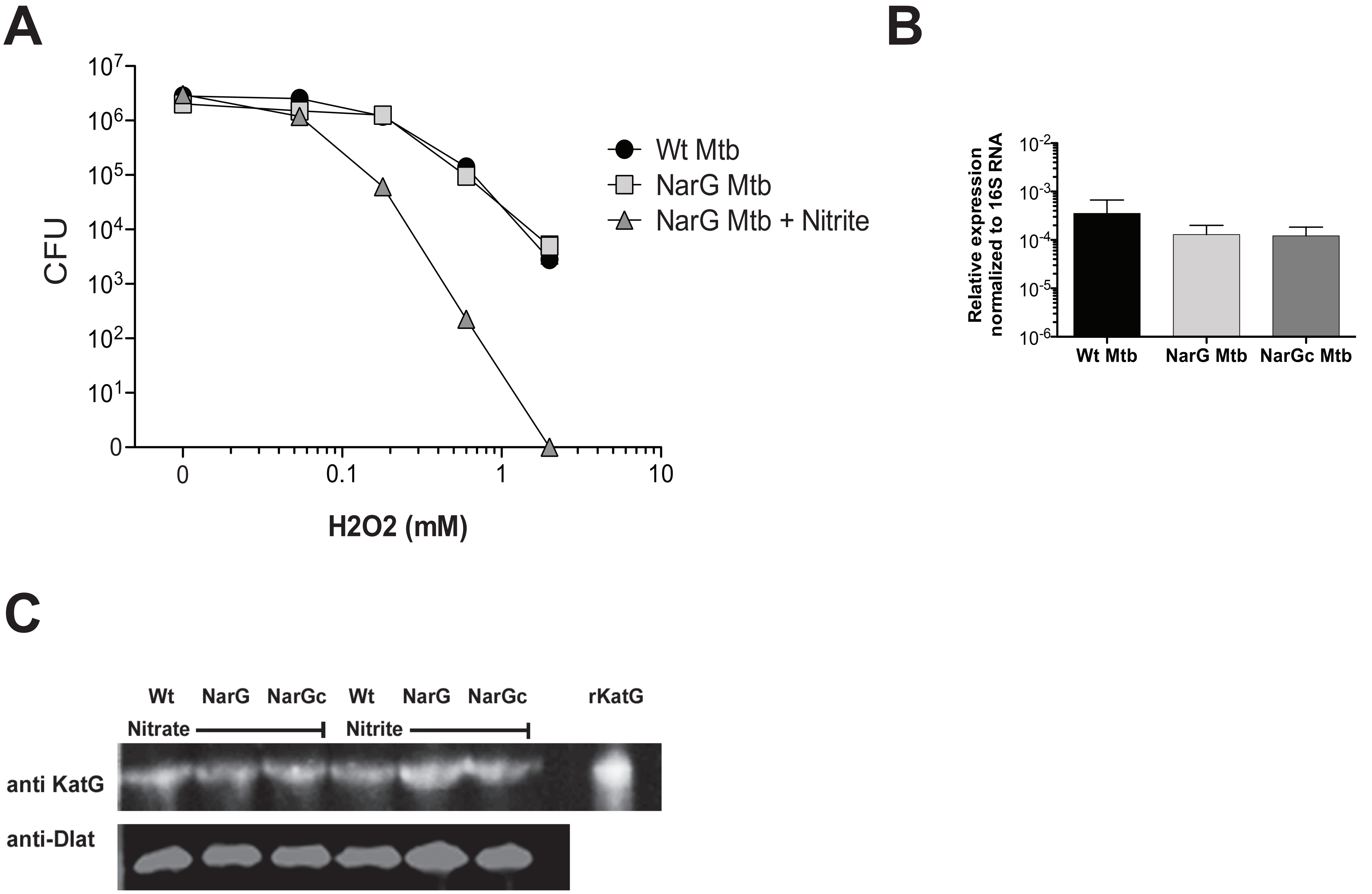

Supplement: Supplementary file 1 [file mbo30002-0901-SD1.pdf]
